# Indonesian martial artists’ preferences in martial arts schools: Sustaining business competitiveness through conjoint analysis

**DOI:** 10.1371/journal.pone.0301229

**Published:** 2024-04-05

**Authors:** Yogi Tri Prasetyo, Maela Madel L. Cahigas, Eugene Patrick, Michael Rodney, Reny Nadlifatin, Satria Fadil Persada

**Affiliations:** 1 International Bachelor Program in Engineering, Yuan Ze University, Chung-Li, Taiwan; 2 Department of Industrial Engineering and Management, Yuan Ze University, Chung-Li, Taiwan; 3 School of Industrial Engineering and Engineering Management, Mapua University, Manila, Philippines; 4 Department of International Business Engineering, Petra Christian University, Surabaya, Indonesia; 5 Department of Information Systems, Institut Teknologi Sepuluh Nopember, Surabaya, Indonesia; 6 Entrepreneurship Department, BINUS Business School Undergraduate Program, Bina Nusantara University, Jakarta, Indonesia; Opole University of Technology: Politechnika Opolska, POLAND

## Abstract

The popularity of martial arts in the present times has become apparent, therefore, it is necessary to explore martial artists’ preferences and the schools’ competitiveness. The purpose of this study was to assess martial artists’ preferences concerning the services offered by martial arts schools. Conjoint analysis was utilized to analyze the responses of fifty-five (55) martial artists based on the seven (7) martial arts schools’ attributes. The results showed that the type of martial arts was found to be the most important attribute (30.449%) followed by distance (27.970%), price range (22.706%), social environment (11.097%), class preference (5.080%), goal (1.562%), and schedule (1.135%). Furthermore, Muay Thai or Kickboxing was the most preferred martial art, Mixed Martial Arts (MMA) was the second priority, next was Taekwondo, then Karate, and finally Boxing. In addition, the martial artists’ preferred distance was less than 8 km, and a monthly training cost of 150,000 to 450,000 IDR (10 to 20 USD). Martial artists liked attending open classes, treated martial arts as a hobby, and favored attending classes once or twice weekly. With the lack of conjoint-related studies in the martial arts industry, the findings contributed to academicians and addressed issues of inadequate studies. Most importantly, the researchers presented managerial implications to leverage marketing techniques and develop the business operations of martial arts schools.

## 1. Introduction

Martial arts allow individuals to use their full body or a specific weapon when applying strike, control, and submission techniques against another person [1, 2]. The most common forms of martial arts include Muay Thai or Kickboxing, Taekwondo, Karate, Wushu, Boxing, Judo or Brazilian jiu-jitsu (BJJ), Pencak Silat, and Mixed Martial Arts (MMA) [2, 3]. In retrospect, these forms were initially learned by a practitioner to protect oneself or brawl against another person [4]. These instances reflect the significance of security among individuals. As the standards of combat sports shift, negative connotations against martial arts violence start to diminish. In today’s society, martial arts were perceived as a self-enhancing activity, physical fitness, leisure sport, and logical competition [1, 4, 5]. They improve human well-being because of the corresponding effects, such as improved posture, relaxed mind and body, and enhanced confidence. Additionally, martial arts can be used to expand one’s social network [6]. In a more comprehensive context, martial art is an instrument to boost the country’s economic conditions. For instance, Thailand’s tourism industry improved by promoting Muay Thai [7]. Foreigners visited the country to learn Muay Thai, watch the actual Muay Thai competitions, and patronize local products and services. Hence, martial arts benefit individuals, stakeholders, and the country’s economy. These motivations behind the increasing popularity of martial arts prompted businessmen to put up martial arts schools.

Martial arts schools are training centers where newbie practitioners and professional martial artists can learn martial arts formally. These formal schools provide practitioners with various combat techniques and nutrition intake under the supervision of coaches. Coaches or trainers guide martial artists holistically because martial arts require physical and mental vitality [3]. Without guidance from licensed instructors and credible martial arts schools, individuals can incur injuries. If individuals are formally trained, accidents can be prevented, especially fatal ones. In Minnesota, United States, martial artists have at least twenty (20) options among the martial arts schools [8]. Similar to Indonesia’s setting, it is necessary to have several options because not all schools offer the same environment and training packages. Martial artists tend to look for variations because they seek skills improvement and training center compatibility [9]. As a consumer, they wanted to satisfy their needs by maximizing the resources of martial arts schools. In a professional setting, police have their own training center because they need to polish their arrest and self-defense skills [10]. But the common martial arts practitioners are hobbyists and competitors [11]. Hence, the demand for martial arts schools in East Java, Indonesia was quite high because of insufficient training centers [12]. In connection with the present study, the majority of volunteer respondents lived in Surabaya, the capital of East Java. This circumstance supports the significance of understanding the business operations of constructing and maintaining martial arts schools within the country. Unfortunately, some martial arts schools struggle due to poor operations management [7].

Managing martial arts schools require continuous operations streamlining to maintain competitiveness. Marketing techniques and operations management signify the branding and stability of martial arts businesses. Nowadays, digital advertisement and word-of-mouth help schools market their identity. Digital advertisements promote the slogans of schools that entice people to try martial arts [11]. Meanwhile, the primary instrument of word-of-mouth is the members of the school or training center. These members encourage co-members to try different types of martial arts and influence their peers to join the martial arts school [6]. Martial arts schools also assess consumers’ leisure behaviors to exploit for profit [13]. Since martial arts are considered a lifestyle, consumers include the sports on their priority list. It should also be noted that the level of commitment to training programs varies for professional athletes and non-professionals [5]. In return, martial arts schools strategically plan their conditioning schedules. Moreover, they utilize action films to capture the attention of newcomers. Some practitioners engaged in martial arts because they were inspired by movie leads [11]. Furthermore, most martial arts schools support televised events, such as Ultimate Fighting Championship (UFC), ONE Championship, and international boxing matches. These events intensify martial arts schools’ engagement with both hobbyists and athletes [8, 14]. The presented business approaches imply that martial arts schools needed regular assessments and developments because consumer behavior frequently changes.

While changes are inevitable, it is plausible to determine martial artists’ preferences in multiple dimensions. Meyer and Bittman [11] identified the Japanese and German practitioners’ motivation to pursue Judo, Taji, Krav Maga, and Wing Chun. They concluded a combination of martial arts influences for each nationality but the most significant ones were gaining ethics, building physical strength, and learning self-defense. Blomqvist Mickelsson [15] assessed the engagement and sociopsychological behavior of youths when practicing MMA and BJJ. BJJ was preferred by youths because it was less aggressive than MMA. It also increased social confidence and respect among practitioners. Moreover, Ong and Ruzmin [6] noted that male Muay Thai practitioners learned the sport for personal achievement but female Muay Thai practitioners liked doing the sport to engage with like-minded martial arts enthusiasts. Although gender is an intriguing issue among martial artists, a study revealed that tenured martial art practitioners adapted to modernity as they disregarded gender and age norms [8]. In past research, veteran martial artists did not have any preferences when attending class sessions. These aforementioned studies exhibited attributes affecting martial artists’ preferences. However, none of those studies evaluated several attributes influencing martial artists’ preferences in martial arts schools.

Martial arts schools provide the needs of hobbyists and competitors by offering conditioning programs, training facilities, and coaches. Green [8] revealed that martial artists were open to training at different schools to enhance their capabilities. This finding showed that competition among martial arts schools was extensive. Kim and Zhang [16] found that benefits and constraints indirectly contribute to martial artists’ satisfaction and commitment to martial arts schools. The past study understood the martial artists’ psychological behaviors and put importance on significant variables using confirmatory factor analysis. Muay Thai schools can also benefit from foreigners as tourism and Muay Thai were strategically connected [7]. Apart from the common martial arts schools, interested practitioners can also learn from educational and rehabilitation establishments [4]. Correspondingly, police have distinct physical training centers where they can practice martial art techniques [10]. Although the past studies delivered the necessary business techniques, none of these presented journals examined direct attributes affecting both martial artists and schools.

Thus, there are limited studies centered on martial artists’ preferences for martial arts schools. Kim and Cruz [17] focused on the martial arts conditioning programs provided by trainers. But Kim and Cruz [17] did not associate the connection among martial artists or consumers, trainers, and martial arts schools. Blue [1] evaluated the martial artists’ experiences in MMA. The past study was fixated on one type of martial art and only translated observations of martial artists qualitatively. Unlike the current study, researchers employed a statistical technique to decipher martial artists’ preferences across different types of martial arts. Another study assessed MMA but positioned its study on forecasting competition results [2]. Although they generated a Markov chain-based model, they overlooked the comparison between hobbyists and competitors. In this study, researchers delve deeper into the importance of martial artists’ goals. Meanwhile, Dongoran et al. [3] catered to the top two goals of martial artists. They assessed the psychological behavior of Indonesian martial artists but only utilized the most basic form of statistics. Normality test, t-test, and descriptive statistics can only interpret variables in a one-way form unlike the multivariate statistical technique utilized by the present research, where relationships among all variables are predicted. Interestingly, Meyer and Bittman [11] scrutinized several types of motives behind participants’ engagement in martial arts. Similar to the previous study, the engagement level of martial artists in attending training at a school during the COVID-19 pandemic was investigated by Monterrosa Quintero et al. [5]. However, motive and engagement level are considered one-sided factors for both studies. They ranked and grouped the respective factors successfully, but overlooked the significance of other factors influencing martial artists’ preferences. In the current study, researchers did not solely focus on one factor. Instead, they considered all factors affecting martial artists and martial arts schools. An effective method to investigate the insights of consumers or martial artists about the business packages offered by martial arts schools is through conjoint analysis.

Conjoint analysis is a well-known statistical approach that identifies consumer preferences by evaluating business attributes [18, 19]. Attributes refer to the value of product or service characteristics [18]. These attributes comprised different sets of levels to scrutinize the fundamental characteristics [18]. Moreover, studies utilized conjoint analysis to leverage business products [20] and services [21] to encourage potential consumers and maintain existing ones. In this study, the services offered by martial arts schools are evaluated. Meanwhile, a few studies utilized conjoint analysis to discuss physical fitness [21–23]. However, regular fitness programs are traditional workouts that assist martial artists to build their strength. They are completely different from martial arts conditioning, especially the intensity and training routine. This argument supports the novelty of the research context and presented methodology.

Therefore, this study aims to analyze martial artists’ preferences towards martial arts schools’ attributes using conjoint analysis. Attributes refer to business packages offered by a standard school. By utilizing conjoint analysis, the researchers assess the attributes affecting the consumers or martial artists. These attributes include the type of martial arts, consumer’s goal, class preference, weekly schedule, school’s distance or location, monthly price range, and social environment. They have underlying levels that determine the appropriate market segmentation. The findings can contribute to the competitiveness of Indonesia’s martial arts schools by applying business practices centered on consumer behavior. Additionally, the business owners, stakeholders, employees, and coaches of martial arts schools can better understand Indonesian martial artists’ behaviors. This result can help Indonesia’s martial arts schools influence newcomers and maintain engagement among existing school members. Finally, this study contributes to the lack of studies focusing on martial artists’ preferences in martial arts schools using conjoint design. Academicians can also utilize the presented discussion in all types of sports, and not only in martial arts.

Overall, the study’s background stemmed from the benefits induced by martial arts, which can be applied by any individual. The readers from the consumer side can resonate with the advantages of physical fitness, memory retention, socialization, and welfare. Meanwhile, readers of the industry or business facet can adopt training, branding, and internal operations strategies. In addition, readers from academia can gain novel knowledge of conjoint analysis in the martial arts industry. These backgrounds and motivations are all directed to sustain the competitiveness among Indonesia’s martial arts schools. Considering these contexts, the succeeding manuscript sections are arranged as follows. Section 2 describes the methods used by researchers by highlighting data collection and conjoint analysis. Section 3 presents the statistical findings. Section 4 explains the presented findings with corresponding academic and practical implications, alongside scope and limitations. Lastly, Section 5 concludes the study’s observations and contributions.

## 2. Methodology

**Fig 1:**
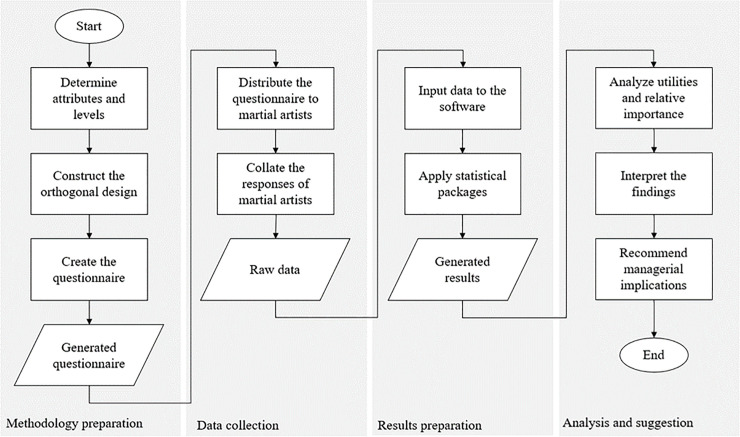
Research framework.

Fig 1 illustrates the chronological order of the study’s experimental design and analysis process. The study’s methodology was prepared by identifying significant martial arts business factors and martial artists’ preferences from relevant studies. These were considered the conjoint design’s attributes and levels. The orthogonal design was constructed by selecting important stimuli and was then transformed into a questionnaire. Next, the data collection occurred as soon as the questionnaire was distributed to Indonesian martial artists. A total of fifty-five (55) Indonesian martial arts practitioners voluntarily participated. They had varying demographics, attribute likings, and level preferences. The respondents’ data was the starting point for results preparation. The researchers maximized IBM SPSS 25 software and employed orthogonal design and syntax conjoint plan package functions. Finally, the generated results were analyzed based on conjoint principles, such as interpreting the most important attributes and levels through utilities and relative importance. The least important attributes and levels were separated. This technique assured that critical attributes and levels were prioritized in presenting recommendations to stakeholders.

Step-by-step details for the study’s methodology were discussed in the succeeding three (3) subsections. The first part tackled the sampling procedure and demographic characteristics of martial artists. The second subsection explained the foundation of establishing a conjoint-based questionnaire. Lastly, the third area discussed statistical techniques incorporated into the data.

### 2.1 Participants

This study was approved by Mapua University and Petra Christian University Research Ethics Committees (FM-RC-22-92). Prior to the data collection, the researchers explained the study’s purpose to each participant. All participants willingly filled out the written online consent form. The data collection were conducted from August 1^st^ 2022 until December 1^st^ 2022.

A purposive sampling technique was applied to gather fifty-five (55) martial arts practitioners. This is the recommended sampling method for studies with pre-determined participants [24]. Since the researchers aimed to analyze the preference of martial artists, all participants must have adequate knowledge and experience. Specifically, the researchers reached out to the members of martial artists’ online groups and well-known martial arts schools in Surabaya, Indonesia. They distributed the questionnaire physically and digitally to respondents who agreed to participate in the study. When participants submitted their responses one at a time, the Kolmogorov-Smirnov test and univariate analysis were continuously applied. These tests supported the significance of a sample size greater than 50 and data with underlying levels for each attribute [3, 25]. Through IBM SPSS 25, the p-value was found statistically significant because all the groupings were less than 0.05. As this procedure was performed during the sampling period, the researchers opted to support the sample size using Kendall’s Tau during the interpretation of the results. It was shown in Table 7 that the participants’ responses were consistent and the sample size was statistically significant at 0.001.

Table 1 represents the demographics of the respondents. Based on the table, more than half (65.5%) of the respondents were male and less than half (34.5%) were female. In addition, the majority of the respondents are young adults with ages between 21 to 25 years old, followed by teenagers (16 to 20 years old), next to the middle age respondents (31 to 45 years old). There were only limited participants from the age brackets of 26 to 30 years old and greater than 45 years old. Meanwhile, a lot of the respondents earned a bachelor’s degree, followed by high school level participants, and master’s degree holders. The participants were allowed to choose multiple options for their martial arts interests. They were mostly interested in Taekwondo (54.5%), Muay Thai or Kickboxing (38.2%), and Mixed Martial Arts (20%). Only a few martial artists were fascinated by Karate, Wushu, Boxing, Pencak Silat, and Judo or Brazilian jiu-jitsu. Finally, the respondents were relatively new to the martial arts world as half of them (50.9%) had less than one year of experience. Nonetheless, veterans who practice martial arts for more than 7 years (18.2%) came second, followed by those with one to three years of experience (16.4%), and three to seven years of experience.

**Table 1:** The demographic profile of participants.

Attribute	Category	Frequency (n)	Percentage (%)
Gender	Male	36	65.5
	Female	19	34.5
Age	16–20 years old	12	21.8
	21–25 years old	38	69.1
	26–30 years old	1	1.8
	31–45 years old	3	5.5
	More than 45 years old	1	1.8
Highest educational attainment	High School level	6	10.9
	Bachelor’s degree	48	87.3
	Master’s degree	1	1.8
Interested in these forms of martial arts	Taekwondo	30	54.5
	Muay Thai/Kickboxing	21	38.2
	Mixed Martial Arts	11	20.0
	Karate	9	16.4
	Wushu	9	16.4
	Boxing	9	16.4
	Pencak Silat	8	14.5
	Judo/Brazilian jiu-jitsu	7	12.7
Years of experience	Less than 1 year	28	50.9
	1–3 years	9	16.4
	3–7 years	8	14.5
	More than 7 years	10	18.2

All participants were briefed about the study’s objectives and filled out a consent form. Lastly, the corresponding author could provide the dataset upon other researchers’ requests. The participant’s personal information, such as full name, residence address, and email address, would not be distributed to protect their privacy.

### 2.2 Conjoint design

The researchers investigated seven (7) martial arts attributes with corresponding levels, as demonstrated in Table 2. The attributes pertained to the type of martial arts (Muay Thai/Kickboxing, Taekwondo, Karate, Wushu, Boxing, BJJ, Pencak Silat, and MMA), goal (competition and hobby), class preference (private and open class), schedule (once or twice a week and more than twice a week), distance (0–8 km, 8–15 km, and more than 15 km), price range (150,000–300,000 IDR, 300,000–450,000 IDR, and more than 450,000 IDR), and social environment (same age only, same gender only, and no preferences).

**Table 2:** Attributes for martial arts training centers.

Attributes	Levels
Type of Martial Arts	Muay Thai/Kickboxing, Taekwondo, Karate, Wushu, Boxing, Judo/BJJ, Pencak Silat, MMA
Goal	Competition, Hobby
Class Preference	Private Class, Open Class
Schedule	Once or twice a week, more than twice a week
Distance	0–8 km, 8–15 km, >15 Km
Price Range	150,000–300,000 IDR, 300,000–450,000 IDR, >450,000 IDR
Social Environment	Same age only, same gender only, no preferences

The first attribute refers to the type of martial arts. It was necessary to identify the participant’s interests since all martial arts have unique movements and focuses. A study noted that martial artists value the type of martial arts because of their physical abilities and health conditions [1]. The common martial arts practiced worldwide are Muay Thai or Kickboxing, Taekwondo, Karate, Wushu, Boxing, Judo or BJJ, Pencak Silat, and MMA [2, 3]. Martial arts practitioners prioritize Muay Thai or Kickboxing, Boxing, and Karate for striking opponents because these techniques develop strong punches and kicks [1]. In Muay Thai, practitioners use fists, elbows, knees, and sheens. Meanwhile, Boxing practitioners can only use their fists. Karate is a Japanese martial art technique allowing both punching and kicking, but it does not allow face punching as it symbolizes disrespect [4]. Meanwhile, Judo or BJJ helps control an opponent through grounding and throwing techniques [1]. MMA is a combination of all these aforementioned techniques [1]. It was deemed physically demanding due to various fighting and defense methods. On the other hand, Taekwondo originated in Korea and Wushu is a Chinese martial art. Both of these are aggressive combat sports because of high-intensity kicks, punches, and holds [3, 26]. While Pencak Silat has similarities to Wushu, it has extreme fierceness because this sport combines weapons alongside close combat techniques.

Secondly, the goal is an attribute evaluated in the study because martial artists establish objectives when committing to martial arts. A past study disclosed that martial artists motivate themselves to participate in martial arts to compete and enjoy [11]. Thus, the researchers recognized competition and hobby as the primary levels of the goal attribute. Martial artists who aim to compete undergo rigorous training because they want to win. They prepare for all kinds of matches by ensuring that attack and defense tactics are incorporated into their strategies [2]. One participant from the study of Blue [1] described that spending more time honing martial arts skills fulfills a part of the person’s goals. While martial arts have professional competitions, a study argued that there were practitioners who treat martial arts as a regular training or hobby [1]. These martial artists were more focused on committing to a healthy lifestyle and finding engaging physical activity. Additionally, some martial artists prefer to use martial arts techniques for personal skill development than applying an aggressive style in a competition setting [11].

The third attribute is the class preference, which reflects the martial artists’ type of learning modes. As people have different likings, one may feel comfortable having skinship with others while others are more reserved [4, 21]. Hence, training centers offer both private and open classes. Private classes are one-on-one training programs between the trainee and the coach. In some fitness centers, private classes can also mean conditioning multiple trainees within a similar circle of friends. But, these private classes have a limited number of attendees and entail a higher cost. Meanwhile, open classes permit all martial artists to join the conditioning program. Open classes may vary with the level of knowledge and expertise. They can be subcategorized as expert and novice classes [4]. This approach ensures that open classes are facilitated among similar levels to help martial arts practitioners adjust easily. Since martial arts require physical training, the practitioners have their preferences when undergoing conditioning programs. Some martial artists are more comfortable with open classes because they are eager to learn with new people [4]. However, COVID-19 affected people’s preferences because combat sports are prone to virus transmission [21]. As a new era started to emerge in the sports industry, people could also form unique habits. This uncertain scenario supported the importance of including class preference in the attribute.

The fourth attribute, schedule, pertains to martial artists’ training frequency. In this study, respondents had diverse age brackets, varying from teenagers to adults. They attend classes depending on their availability, especially since they have school or work commitments [1]. Due to this scheduling concern, martial artists prepare different sets of schedules and training programs. Martial artists opt to train depending on their condition and availability, which can be once, twice, or several times a week [10]. Their best condition could be attained by attending classes consistently. Considering the relevant studies, the current study analyzed once or twice a week and more than twice a week for participants’ scheduling preferences. Moreover, it was noted that class frequency and type of martial arts were associated with each other [10]. Some forms of martial arts required intense training while others did not demand meticulous preparation. One of the past studies also noted that 24-hour fitness gyms were marketable for both office workers and students [21]. Although these fitness centers offered martial arts programs, martial artists encountered scheduling issues with coaches. Martial arts practitioners were dependent on coaches. These coaches spearheaded conditioning programs and provided emotional support [26]. Therefore, the schedule attribute was connected with several factors before martial artists could decide on the class frequency.

Next, the distance attribute applies to the proximity preferences of respondents. Distance had a great influence on gym-goers because of convenience [27]. In the current study, the researchers considered 0 to 8 kilometers, 8 to 15 kilometers, and more than 15 kilometers distance from participants’ residences to the martial arts school. These distances were considered using the location of common training centers in Indonesia. Particularly, a distance of 0 to 8 km is walkable or can be driven for 20 to 30 minutes. Meanwhile, 8 to 15 km can be reached anywhere from a 31- to 40-minute drive. Lastly, more than 15 km is attainable by driving for at least 41 minutes. These minutes were estimated without heavy traffic conditions. It should be noted that traffic is inevitable when living in a metropolitan area. On rare occasions, a few kilometers of distance can take an hour if drivers encounter traffic congestion. Nevertheless, some MMA practitioners were willing to go on an approximate 45-minute drive [8]. In comparison to the present methodology, a 45-minute drive was linked with the farthest distance level.

Furthermore, the price range attribute considers the financial capability of martial artists. The typical martial arts school in Indonesia offers the following price range every month: 150,000 to 300,000 IDR (10 to 20 USD), 300,000 to 450,000 IDR (21 to 30 USD), and more than 450,000 IDR (at least 31 USD). These approximate price ranges were assessed in the conjoint design’s levels. One month’s session may entail two to four classes, depending on the school protocols. In the United States, training for martial arts was a fringe benefit to wealthy people because regular training programs were costly [8]. Thus, the price range must consider the average salary of employees and the allowance of students. In view of their willingness to spend, many would consider going to martial arts school if the price was affordable. For instance, Philippine gym-goers preferred fitness centers that charged at most 20 USD [21]. However, they also valued coach services and amenities on top of the price range.

Lastly, the social environment is an essential attribute because martial art is known for its masculinity concept. Both gender and age were considered because human norms and training center practices change. For instance, the participants’ principles in one class might be different from another, and martial arts’ techniques and equipment may vary for all martial arts centers [27]. It was also noted that age and gender were the common demographic characteristics noticed by gym members [1]. Thus, the study considered the following social environment levels: same age only, same gender only, and no preferences. One study noted that most martial artists were on the older side compared to the younger ones [10]. But another study argued that age was noteworthy to compare when attending professional training programs [26]. Although martial arts were starting to entice women, it was concluded that the sports were dominated by males [8]. Thus, training centers typically offered women-to-women classes (all women participants including coaches), men-to-men classes (all men participants including coaches), and no gender difference (mix of women and men in one class). Some preferred attending training with both genders, others felt more comfortable in same-gender glasses, and a few did not mind gender differences [27].

Table 3 demonstrates the synopsis of methods utilized by past studies. These researchers considered a variety of martial arts schools’ attributes and martial artists’ focuses. It could be depicted that past studies explored different statistical techniques, optimization strategies, and experimental designs. Unfortunately, only one research utilized conjoint analysis. But this study did not cover an in-depth analysis of fitness practitioners as it was centered on all kinds of gym-goers [21]. Table 3 also shows that the attributes and focuses of the current study were more comprehensive compared to the existing ones. In this study, the researchers covered comprehensive conjoint analysis to cater to a wide array of martial arts schools’ target markets.

**Table 3:** Comparison of methods.

References	Highlighted Attributes	Focus of Practitioners	Methods
Blue [1]	Types of martial arts, goal, schedule, and social environment	MMA	Direct observations based on carnal ethnography and rhythmanalysis
Holmes et al. [2]	Types of martial arts	MMA	Markov chain model
Dongoran et al. [3]	Types of martial arts	Taekwondo, Karate, Wushu, Pencak Silat, Boxing, and Judo	Ex post facto with a retrospective causal-comparative design
Harwood-Gross et al. [4]	Types of martial arts and class preference	Dennis Survival Jujitsu (DSJJ)	Linear Mixed Model (LMM) and post-hoc analyses with Bonferroni correction
Green [8]	Distance and price range	MMA	Interview responses analysis
Renden et al. [10]	Schedule and social environment	Kickboxing, Karate, Jiu-Jitsu, and Krav Maga	Experimental design, descriptive statistics, and ANOVA
Meyer & Bittman [11]	Goal	German and Japanese Karateka	Descriptive statistics
Ong et al. [21]	Class preference, schedule, and price range	All kinds of gym users	Conjoint analysis
Mehrsafar et al. [26]	Types of martial arts, schedule, and social environment	Wushu	Repeated measures ANOVA
Jansson et al. [27]	Distance and social environment	Outdoor gym users	Historical analysis of peer-reviewed journals

### 2.3 Statistical analysis

Conjoint analysis is a multivariate statistical technique that interprets consumer preferences by identifying the observed product or service attributes [28]. These attributes have underlying levels, marketable to any type of consumer. This statistical technique’s major advantage is the reliability of results despite the limited number of samples [18]. The study gathered fifty-five (55) participants and this number was relatively small. Nevertheless, Guo et al. [18] noted that experiments with minimal samples could still be performed. In addition, the researchers utilized conjoint analysis because it could translate numerical data as a response to real-world problems [29]. Therefore, the conjoint analysis was used to analyze martial artists’ responses to the questionnaire, especially in interpreting a combination of their priorities.

IBM SPSS 25 was utilized to run the conjoint analysis with an orthogonal design. The orthogonal method generates stimuli, which represent a series of levels across all attributes [18]. A total of twenty-one (21) stimuli were generated as presented in Table 4. Respondents evaluated all stimuli using a 7-point Likert scale. They were asked, “On a scale of 1 (strongly dislike) to 7 (strongly like), how much do you dislike or like the following combinations?” These 21 stimuli acted as the primary questionnaire of the experimental conjoint analysis. In addition, two (2) holdouts were added to test the consistency of the respondents. These holdouts ensured data reliability [21].

**Table 4:** Twenty-one stimuli of the conjoint design.

Combination	Type of Martial Arts	Goal	Class Preference	Schedule	Distance	Price Range	Social Environment
1	Taekwondo	Competition	Open Class	Once or twice a week	8–15 km	300,000–450,000 IDR	Same age only
2	Muay Thai/Kickboxing	Competition	Open Class	More than twice a week	8–15 km	>450,000 IDR	No preferences
3	Karate	Hobby	Open Class	Once or twice a week	8–15 km	150,000–300,000 IDR	Same gender only
4	Taekwondo	Competition	Private Class	More than twice a week	>15 Km	150,000–300,000 IDR	No preferences
5	Pencak Silat	Competition	Open Class	Once or twice a week	0–8 km	>450,000 IDR	Same gender only
6	MMA	Competition	Open Class	Once or twice a week	0–8 km	300,000–450,000 IDR	No preferences
7	Muay Thai/Kickboxing	Hobby	Open Class	More than twice a week	0–8 km	150,000–300,000 IDR	Same age only
8	Boxing	Competition	Private Class	Once or twice a week	0–8 km	150,000–300,000 IDR	Same age only
9	Pencak Silat	Competition	Private Class	Once or twice a week	>15 Km	150,000–300,000 IDR	No preferences
10	MMA	Competition	Private Class	Once or twice a week	8–15 km	150,000–300,000 IDR	Same gender only
11	Boxing	Hobby	Private Class	Once or twice a week	8–15 km	>450,000 IDR	No preferences
12	Muay Thai/Kickboxing	Competition	Private Class	Once or twice a week	8–15 km	>450,000 IDR	No preferences
13	Wushu	Hobby	Private Class	Once or twice a week	0–8 km	300,000–450,000 IDR	No preferences
14	Muay Thai/Kickboxing	Competition	Private Class	Once or twice a week	0–8 km	150,000–300,000 IDR	Same age only
15	Wushu	Competition	Private Class	More than twice a week	8–15 km	150,000–300,000 IDR	Same gender only
16	Taekwondo	Hobby	Open Class	Once or twice a week	8–15 km	150,000–300,000 IDR	No preferences
17	Taekwondo	Hobby	Private Class	Once or twice a week	0–8 km	>450,000 IDR	Same gender only
18	Muay Thai/Kickboxing	Hobby	Private Class	Once or twice a week	>15 Km	300,000–450,000 IDR	Same gender only
19	Muay Thai/Kickboxing	Competition	Private Class	Once or twice a week	>15 Km	300,000–450,000 IDR	Same gender only
20	Judo/BJJ	Hobby	Open Class	Once or twice a week	>15 Km	150,000–300,000 IDR	No preferences
21	Karate	Competition	Private Class	Once or twice a week	>15 Km	>450,000 IDR	Same age only

**Fig 2:**
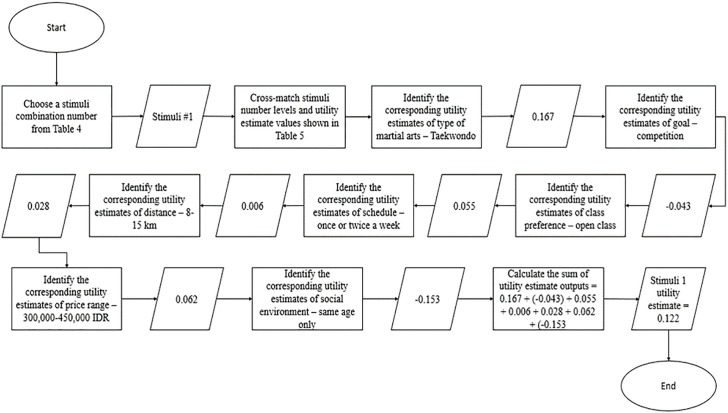
Stimuli number 1 grading guidelines.

An example of stimuli grading was displayed in Fig 2. Firstly, each stimuli combination was graded one at a time as they comprised different attribute levels. Once a stimuli number was selected, the corresponding values under the utility estimate column presented in the results section (Table 5) should be complemented. This procedure was repeated seven times since the study evaluated seven attributes. Then, the recorded individual utility estimate values were all added. The total value represented the stimuli number’s final utility estimate.

**Table 5:** Attribute and level preferences.

Attribute	Level	Utility Estimate	Standard Error	Average Importance Value (%)
Type of Martial Arts	Muay Thai/Kickboxing	0.355	0.076	30.449
	Taekwondo	0.167	0.094	
	Karate	0.115	0.122	
	Wushu	-0.301	0.094	
	Boxing	0.074	0.131	
	Judo/BJJ	-0.207	0.119	
	Pencak Silat	-0.446	0.142	
	MMA	0.243	0.134	
Goal	Competition	-0.043	0.051	1.562
	Hobby	0.043	0.051	
Class Preference	Private Class	-0.055	0.046	5.080
	Open Class	0.055	0.046	
Schedule	Once or twice a week	0.006	0.055	1.136
	More than twice a week	-0.006	0.055	
Distance	0–8 km	0.373	0.057	27.970
	8–15 km	0.028	0.063	
	>15 Km	-0.401	0.057	
Price Range	150,000–300,000 IDR	0.303	0.056	22.706
	300,000–450,000 IDR	0.062	0.069	
	>450,000 IDR	-0.365	0.054	
Social Environment	Same age only	-0.153	0.082	11.097
	Same gender only	-0.189	0.061	
	No preferences	0.342	0.057	
(Constants)		3.381	0.068	

## 3. Results

Table 5 displays the utility estimates and importance values of the conjoint experimental design. The average importance values for each attribute indicated the magnitude of the attribute’s value as evaluated by respondents. Hence, a higher percentage value was the most preferred attribute, and respondents’ liking weakened as the percentage decreased [31]. Based on the presented results, the most important to least important attributes for the respondents were organized as follows: type of martial arts (30.449%), distance (27.970%), price range (22.706%), social environment (11.097%), class preference (5.080%), individual’s goal (1.562%), and schedule (1.136%).

Meanwhile, the utility estimate described the respondents’ attribute level preferences. Positive and high utility estimates depicted that the attribute level was preferred over negative and low values [21]. The total utility value was zero when level utility estimates across each attribute were added. There should be zero-centered differences to ensure preference data accuracy [30]. Among all the levels under the type of martial arts, respondents favored Muay Thai or Kickboxing, followed by MMA, Taekwondo, Karate, and Boxing. Most respondents engaged in martial arts to maintain it as a hobby than the intention to compete. Martial artists liked open than private sessions with coaches. They preferred attending classes once or twice a week to more than twice a week. Next, a lot of respondents gave importance to short distances from residence to martial arts school; specifically, 0 to 8 km was the most important followed by 8 to 15 km distance. For the price range, most of them chose the cheapest (150,000–300,000 IDR), next to 300,000–450,000 IDR. Surprisingly, a lot of the participants did not prefer any age and gender within the training center’s social environment.

All twenty-one (21) stimuli underwent the ranking process by adding their respective attribute level’s utility estimates. Table 6 shows the total utility estimate of each combination and the stimuli’s corresponding rank. It was distinguished that the top five stimuli combinations were arranged as follows: the 6^th^ combination ranked first, followed by the 7^th^ combination, then the 16^th^ combination, next was the 14^th^ combination, and the fifth spot went to the 8^th^ combination. To highlight the stimuli combination that ranked first, the 6^th^ combination’s attribute levels consisted of MMA, competition, open class, once or twice a week, 0–8 km, 300,000–450,000 IDR, and no preferences. On the other hand, the least preferred group was the stimuli combination number 21. This combination entailed the following attribute levels: Karate, competition, private class, once or twice a week, >15 Km, >450,000 IDR, and same age only.

**Table 6:** Stimuli ranking.

Stimuli Combination	Utility Estimate’s Total	Rank
1	0.122	13
2	0.366	7
3	0.361	8
4	0.307	9
5	-0.609	20
6	1.038	1
7	0.970	2
8	0.505	5
9	-0.294	19
10	0.293	10
11	0.073	14
12	0.268	11
13	0.470	6
14	0.786	4
15	-0.263	17
16	0.944	3
17	-0.020	15
18	-0.179	16
19	-0.265	18
20	0.141	12
21	-0.896	21

Table 7 denotes the generated values using the correlation technique. The Pearson’s correlation coefficient (r) value was 0.991, which signified an almost perfect positive linear relationship among observed variables. A value closer to 1.00 was deemed to have a strong relationship [20]. Moreover, Pearson’s r was significant at 0.001, which supported the result’s acceptability. Furthermore, Kendall’s Tau value was 0.900 and it had a 0.70 cut-off as disclosed by Ong et al. [31]. A value of 1.000 for Kendall’s Tau for holdouts was also considered adequate [31]. These findings depicted satisfactory data from respondents because they ensure data consistency.

**Table 7:** Statistical identifiers using correlation approach.

Statistical Analysis	Value	Significance
Pearson’s correlation coefficient	0.991	0.001
Kendall’s Tau	0.900	0.001
Kendall’s Tau for Holdouts	1.000	

## 4. Discussion

### 4.1 Interpretation of conjoint analysis results

The study’s purpose was to investigate martial artists’ preferences in choosing martial arts school’s attributes. This aim was achieved by feeding the collated data to the conjoint method. Also, it was further supported by the application of other statistical techniques, such as preference ranking, utility estimates, Pearson’s correlation, and Kendall’s Tau.

**Fig 3:**
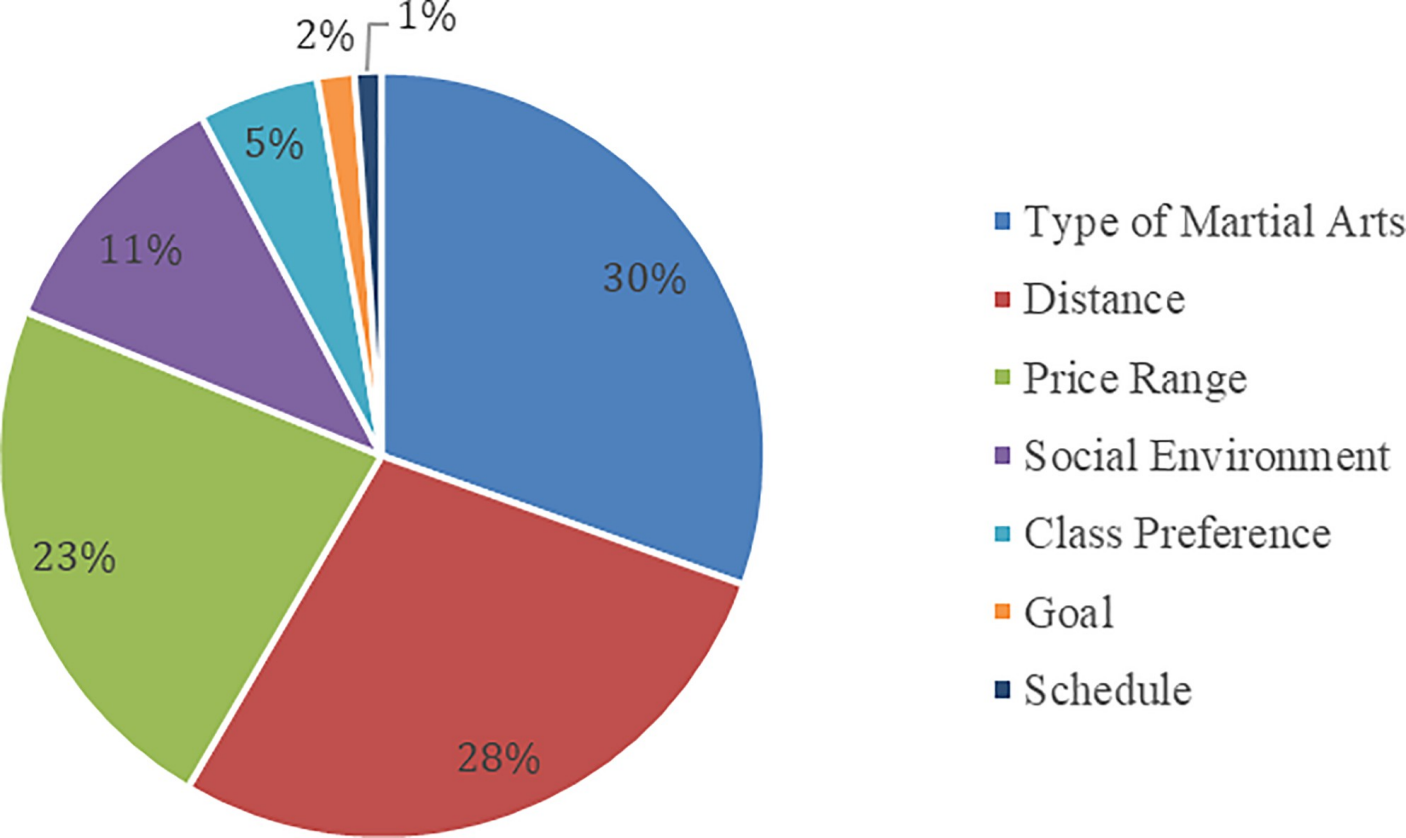
Attribute importance value.

Among the seven (7) conjoint attributes in Fig 3, the type of martial arts produced the highest importance value (30.449%). The respondents chose a combination by putting greater importance on the type of martial arts when going to the training center. For example, respondents who liked Muay Thai or Kickboxing would choose options containing this keyword before considering other attributes and levels. They needed to ponder on the type of martial arts because intensity levels were comparable [4]. Martial artists knew their limits alongside the help of their coaches. Thus, coaches strategize conditioning programs to mitigate physical and mental health risks [3]. These possible issues may occur because martial arts are combat sports that might inflict physical injuries and emotional distress. In a similar context, the type of physical exercise mattered because of the equipment needed to train martial artists [27]. For instance, MMA requires more intensive training and a wide array of gym equipment than Kickboxing [8]. If martial arts school would not offer the equipment needed for a certain martial art, it could affect martial artists’ interests in pursuing the combat sport. These were the primary reasons behind prioritizing the type of martial arts over other attributes. In comparison to past studies, the study of Jansson et al. [27] focused on outdoor exercises, unlike the present study which evaluated both indoor and outdoor physical exercises. Meanwhile, Green [8] overlooked other types of martial arts and only investigated MMA; thus, the current approach was considered more novel by scrutinizing eight (8) types of marital arts.

**Fig 4:**
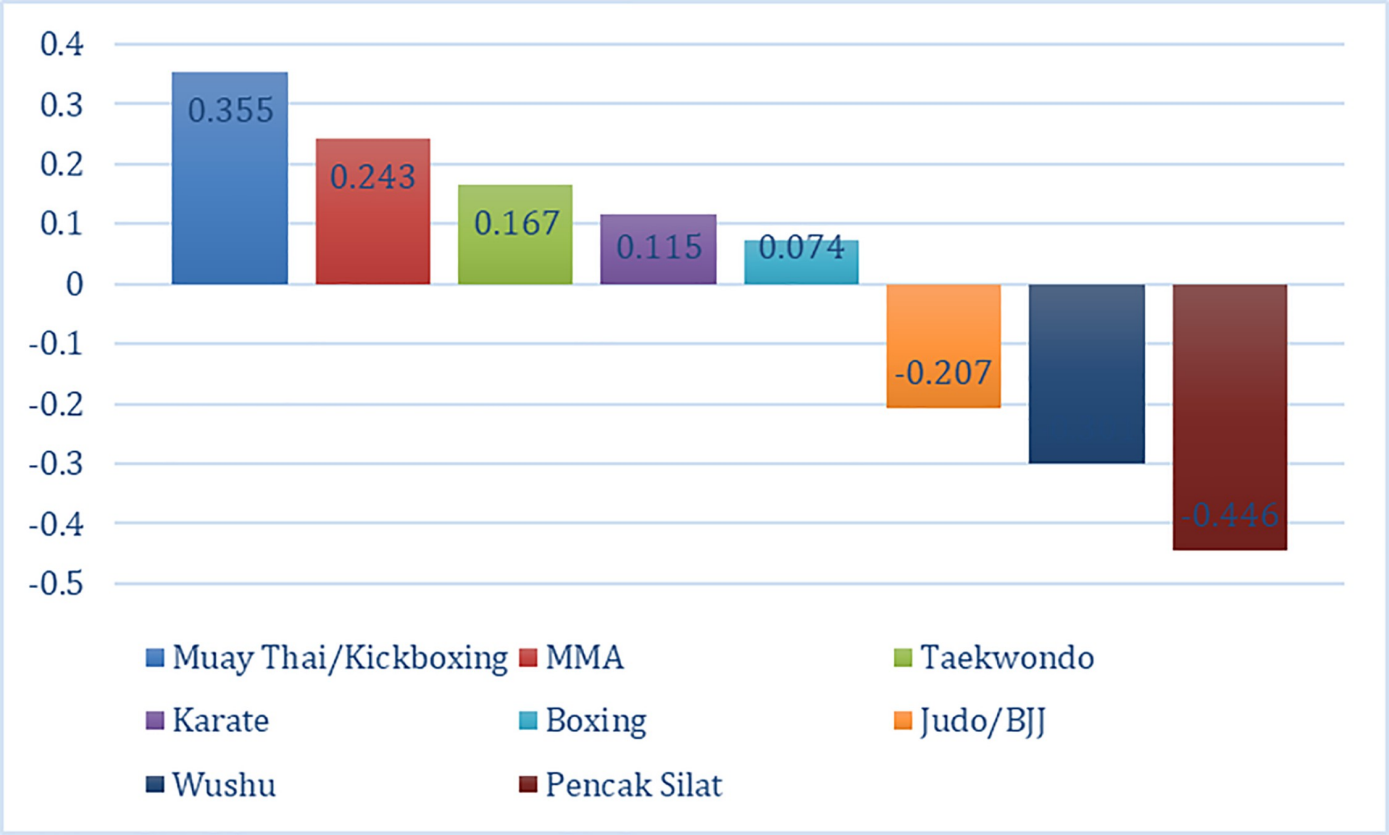
Utility estimates of the type of martial arts.

Fig 4 illustrates the most to the least preferred type of martial arts considering the eight (8) levels: Muay Thai or Kickboxing (0.355), MMA (0.243), Taekwondo (0.167), Karate (0.115), Boxing (0.074), Judo or BJJ (-0.207), Wushu (-0.301), and Pencak Silat (-0.446). Muay Thai or Kickboxing dominated the list because of its popularity across the world. Although it originated in Thailand, several countries adopted this martial art because of its integrated discipline [7]. This combat sport has been featured in movies, TV shows, video games, and many more platforms. Moreover, most respondents reside in Surabaya, Indonesia, and thirteen (13) martial arts schools offered Muay Thai or Kickboxing within the city. The geographical location presented a unique result, which was never discussed in past studies. On a macro-level analysis, there were one million professional Muay Thai practitioners worldwide [6]. This number was expected to increase as the past study did not include non-members of the Muay Thai Kickboxing Federation. MMA ranked second among the type of martial arts levels and its intensity is incomparable to Muay Thai. Muay Thai outweighed MMA because MMA was known for controversies [8]. MMA is prone to violence, which affected the perceptions of martial artists. Findings showed that not everyone preferred an intense combat sport. Martial artists needed to learn a combination of fighting and defense moves from different combat sports to master MMA [1]. Its market is centered on young people as they are more open to learning MMA [2]. The past study’s target coincided with the current study’s demographic characteristics since most respondents were relatively young (21 to 25 years old). Although MMA has negative connotations, it offers unique conditioning and competition approach compared to Boxing [2]. A past study stated that practitioners preferred MMA to Boxing [2]. Likewise, the current study revealed that MMA had a higher level of preference than Boxing.

Furthermore, martial artists’ 3^rd^ to 5^th^ martial arts preferences were Taekwondo, Karate, and Boxing, respectively. Average aggressiveness was essential in Taekwondo and Boxing, but these sports were less aggressive than MMA [3]. In this study, MMA was still preferred despite its high-intensity training program. However, Muay Thai had a lesser intensity compared to MMA, and Muay Thai was the most preferred combat sport. This domino result reflected that the participants did not choose the type of martial arts based on the intensity level. Surprisingly, Taekwondo practitioners with at least four years of experience kept their interest in the sport compared to those with less than four years of experience [11]. Since most respondents had less than one year of experience in Taekwondo, it affected Taekwondo’s ranking level. Nevertheless, the result was deemed promising because the respondents did not completely disregard Taekwondo. This implied that Taekwondo is one of the well-rounded sports in the martial arts industry, which other studies failed to highlight. Meanwhile, Karate is a children-friendly sport and is more accepting of women [8]. As this placed fourth in martial arts preference, it meant that participants valued inclusive combat sports. Muay Thai entails a greater risk of injury than Karate but has a lesser risk than MMA and Boxing [14]. Thus, Karate was the safest sport among all these types of martial arts that scored positively in utility estimate. Although Boxing is one of the common sports, it only placed fifth and had the least positive utility estimate. Practitioners still liked it because it is an enjoyable sport, but they tend to lose interest due to the rise of contemporary sports. This argument was supported by Blue [1] as the researcher found some boxers who would opt to master MMA after trying Boxing. Another reason was the associated social class when playing a sport. Green [8] considered Boxing a low-class sport because it was more common in the marginalized community [8]. Participants of the current study prioritized sports with a high-class tagline and a more established system. Given all these aforementioned factors in choosing the type of martial arts, martial artists considered professionalism, practical values, popularity, Judo or BJJ, Wushu, and Pencak Silat had negative utility estimates, which exhibited that respondents deprioritized these martial arts. Judo or BJJ was one of the martial sports that would inflict high chances of injury alongside Muay Thai, Karate, and Boxing [14]. However, the latter three martial arts generated positive utility estimates compared to Judo or BJJ. Thus, the researchers argued that respondents deprioritized Judo or BJJ, not because of safety issues. Particularly, respondents aimed to steer away from traditional martial arts. Judo or BJJ was included as one of the most traditional martial arts practiced worldwide [4]. Analogous to Wushu and Pencak Silat, martial artists yearn for non-conventional teachings and physical activities. Unfortunately, Judo or BJJ, Wushu, and Pencak Silat lacked modern equipment and techniques. The psychological state and social environment were other factors that affected respondents’ least preferences for Wushu. Apparently, Wushu was perceived to be a catalyst of stress because it entailed full-body contact [26]. A study also revealed that Wushu practitioners were only interested in the sport because of their peers [11]. Hence, the researchers concluded that Wushu was not an ideal sport for an individual pursuer. In addition, martial artists lacked interest in trying Pencak Silat although the study was conducted in Indonesia, where Pencak Silat originated. This circumstance showed that Pencak Silat was unpopular even among Indonesians. The current study’s statement was supported by Moser [13] because Indonesians rarely gave importance to promoting the sport due to the lack of government support. While Pencak Silat was deemed popular around Southeast Asia, it was not as engaging as other martial arts.

Goal attribute (1.562%) only ranked sixth among all seven attributes. This result demonstrated that martial artists were least concerned about their motives when participating in any martial arts. Respondents did not mind learning a specific martial art to compete or enjoy. On a similar note, the goal was not one of the important factors because it could easily be changed [11]. The past study concluded that a competitive stance could be changed into a hobbyist and vice-versa, because of external impulses, insights, and social changes. At a certain point, martial artists wanted to gauge their capabilities by joining a competition. But in this study, most respondents treated martial arts as a hobby than a competition. Both international and local competitions would trigger stress [26], which martial artists in the present study did not prefer to experience. Most practitioners valued affiliation with reputable training centers to help them achieve their fitness goals over engaging in competition [6]. Contrary to the belief that practitioners learn martial arts to compete [10], the current findings argued that respondents participate in training programs to develop a hobby. Martial hobbyists engage in martial arts to improve their confidence, maintain a healthy lifestyle, maximize spare time, and find an enjoyable sport.

In a comprehensive approach, Muay Thai and MMA were both familiar to competitors and hobbyists [8, 14]. However, these studies failed to recognize which goal was more dominant. Hence, the current study concluded that martial artists preferred hobby over the competition goal. Likewise, only a few practitioners competed in MMA because it required full-time commitment and rigorous training [1]. Interestingly, Taekwondo practitioners were motivated to complete training programs to please their parents [11]. This could be comparable to the current study because young adults were more dependent on their parents compared to the older generations. It implied an underlying hobby goal since parents wanted their children to instill discipline. Meanwhile, Karate practitioners’ goal of pursuing martial arts was due to curiosity [11]. They wanted to try new activities during their leisure time. A researcher interviewed participants and revealed that Boxing was regarded as a hobby because it was a fun sport [1]. These presented studies coincided with the result of the current research. Most of the preferred types of martial arts coincided with martial artists’ favored goal (hobby). These discussions debunked the connotation of competition among martial arts practitioners. The researchers brought a new idea of exploring different goal contexts, apart from comparing the typical hobby and competition goals.

The class preference did not garner a high-ranking place either. It only ranked fifth with an importance score of 5.080%. Since class preference was included in the bottom three conjoint attributes, martial artists were least concerned about attending private and open classes. Nevertheless, respondents would opt for an open session than private training. The identified martial arts required constant interaction with coaches and other practitioners to increase their knowledge and techniques. Blue [1] and Ong and Ruzmin [6] discoursed that best health practices and martial arts techniques were adopted by joining different groups. These groups encouraged martial artists and assist with their physical and mental health. Group or open classes would include mindfulness-based intervention to help practitioners train their minds, mitigate stress, and balance their well-being [26]. This open discussion and group support were the primary factors for prioritizing open classes. However, a study concluded that gym-goers preferred one-on-one training to private sessions [21]. Result differences occurred due to the depth of the training program. Ong et al. [21] investigated regular fitness while the current study assessed martial arts. Thus, the study argued that participants liked private classes for regular fitness training programs while open classes would produce more advantages for martial artists’ conditioning. Another contradicting result was also presented by Meyer and Bittman [11]. They found that most martial artists had the least interest in attending open sessions. Since the past study evaluated the perceptions of Germans and Japanese, the Indonesian demographics drew class preference differences. It could be noted that Indonesians were more accepting of group classes as they were more eager to learn from others’ experiences. The presence of COVID-19 also affected the sensitivities of martial artists because they became wary of their surroundings during this period [5]. But the study showed that COVID-19 was not a deciding factor for Indonesian martial artists. As of this writing, Indonesia surpassed the peak of the pandemic and cases started to decrease. It could be implied that training centers conscientiously applied COVID-19 protocols and martial artists were unbothered by the potential transmission.

The schedule (1.136%) was the least preferred attribute among the seven attributes. Martial artists would prioritize all the other attributes before considering scheduling options. This discovery represented martial artists’ flexibility when attending training sessions. It was also noted that none of the conjoint-related studies assessed schedule, resulting in the lack of benchmark [21–23]. Hence, the present findings contributed to the unique implementation of the conjoint design. Mehrsafar [26] encouraged practitioners to follow the designated training programs prepared by training centers. The respondents could easily follow these programs because of their flexibility. But most Indonesians had issues with office demands and failing to attend classes regularly [13]. This present research argued that respondents’ age ranges were mostly linked with students and entry-level employed individuals. It demonstrated that students and fresh graduates could quickly free up their schedules to make time for martial arts training. Among the two levels under the schedule attribute, most respondents liked attending classes once or twice a week compared to more than twice a week. This finding coincided with the goal attribute because most martial artists were considered hobbyists. Blue [1] noted that daily training for eight weeks was only needed for participants who wish to join competitions. Similarly, respondents personally uttered training for six to ten hours a week and more than twice a week to prepare for local competitions [8]. Training at least three times a week helped martial artists understand techniques quickly because constant repetition would increase muscle memory [1]. The demographics showed that most martial artists had less than a year of experience, which was deemed a novice in the field. These beginners could still maximize their potential by training once a week [10]. Since the study gathered enough respondents practicing martial arts for more than seven years, a schedule of once or twice a week was still considered appropriate. This was supported by Harwood-Gross et al. [4], as they noted that expertise could be disregarded and they recommended martial artists to attend training twice a week. As presented in the conjoint result, once or twice a week of training was enough for most participants.

**Fig 5:**
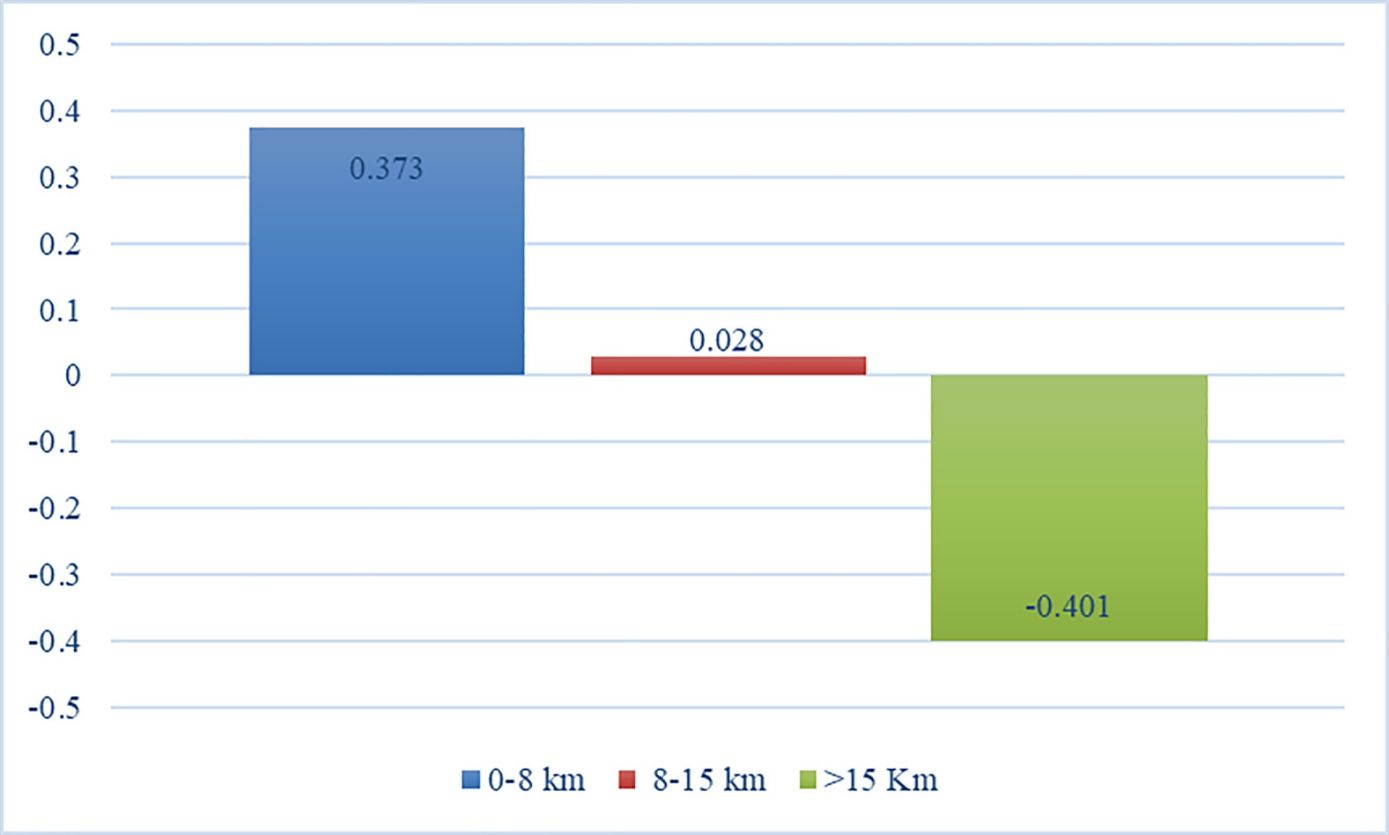
Utility estimates of the distance.

Surprisingly, as presented in Fig 5, distance was found to be the second most important attribute (27.97%). After considering the type of martial arts, respondents would put importance on the training center’s distance from their residences. Most of the respondents lived in Surabaya, Indonesia, which is a high-density place. They valued the training center’s location to minimize unproductive hours spent on travel time. Therefore, martial artists preferred the least distance (0 to 8 km), followed by the average distance (8 to 15 km), and the least preferred was the farthest distance (> 15 km). Jansson et al. [27] tabulated several studies and exposed that practitioners would look for training centers within 2 kilometers of their homes. The presented distance yielded within the least distance (0 to 8 km) as evaluated in the current study. However, a study noted that some martial artists did not mind exerting an effort to visit a training center with more than 15 km distance from home [8]. Contrasting results were evident due to geographical’s urban transportation. Green [8] evaluated martial artists’ preferences from the U.S., where road condition was better compared to Indonesia. Thus, Indonesian martial artists considered traffic conditions when they preferred the 0 to 8 km over other attribute levels. This inference was overlooked by researchers that evaluated Indonesia’s physical fitness industry [12, 13], which added originality to the present study.

**Fig 6:**
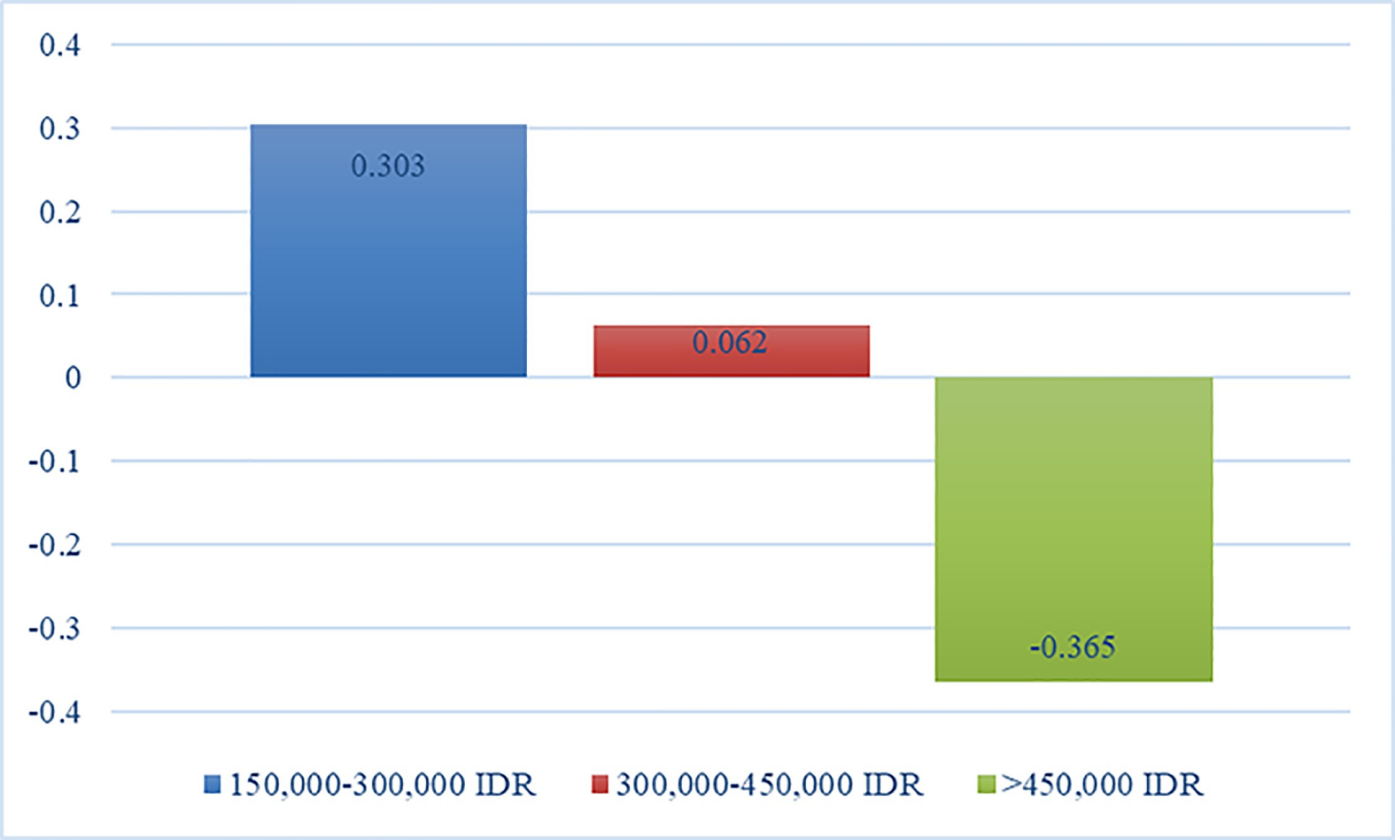
Utility estimates of the price range.

The price range ranked the third most essential attribute with an importance value of 22.706% (Fig 6). The majority of the respondents were young adults who were students, fresh graduates, and entry-level employees with limited financial resources. It was understandable for them to consider martial arts’ price packages. Another study assessed the price preference of gym-goers and identified it as the top attribute when enrolling in a fitness center [21]. Parallel results occurred since the past and current studies generated analogous respondents’ demographic characteristics. Additionally, the price was subtly associated with class preference and schedule because cost packages would vary on the martial artists’ appointments at the training center [27]. The researchers previously presented that respondents preferred open classes and the least training frequency. These features coincided with the least price range because they offered a lesser amount compared to private classes and training programs with several visits. Particularly, respondents found the least monthly package (150,000 to 300,000 IDR or 10 to 20 USD) as the most reasonable and preferred price. The second priority level was an average of 300,000 to 450,000 IDR (21 to 30 USD) price range. Ong et al. [21] yielded the same results as most gym-goers preferred at most 20 USD training price, next to 21 to 41.50 USD. The past study evaluated gym-goers in the Philippines and the country had an almost similar economic condition to Indonesia. Meanwhile, martial artists in the U.S. were willing to consider monthly fees of 99 to 200 USD [8]. This high-priced amount was not preferred by the current respondents as they had negative utility estimates for the highest price range (450,000 IDR or at least 31 USD). Hence, the researchers declared that employees’ starting salaries and students’ allowances played a crucial role. It was vital to fulfill martial artists’ interests by not spending a huge amount. Since there were several options in Indonesia, martial artists could pick training centers that offered affordable martial arts packages.

The social environment got the 4^th^ highest-ranking attribute (11.097%). Interestingly, most respondents put high importance on no age and gender preferences. Meanwhile, the same age and same gender got negative utility estimates. Between these two, the same gender was the least preferred because it had the largest negative value. It was initially assumed that some people might feel uncomfortable when they had to perform combat sports with other people. This assumption was strengthened by a study where female martial artists reduced skinship with other athletes [5]. However, the current study’s assumptions and the past study’s results were debunked. These remarks supported the importance of continuous research as marital arts practitioners were inclined to change preferences as a consumer. Additionally, one of the compelling reasons was having male-dominated research respondents. Male participants were unconcerned about the gender and age of participants in a similar training program. They liked joining mixed groups to learn new techniques from all walks of life. Likewise, several studies dominated by male respondents noted that they preferred training with a diverse group of strangers, so they could further their capabilities [1, 8, 11]. Some martial artists also thought it would be good to train with others to gauge their ability levels [14]. Hence, martial artists valued learnings more than feeling self-conscious. Age and gender were only significant for outdoor sports [27]. Outdoor sports were visible to non-joiners while indoor sports could only be accessed by gym members. The participants’ level of consciousness was manageable every time they interacted with people from the training center compared to being seen by unknown people. In this study, the researchers evaluated martial art programs facilitated inside a training center.

Considering all the itemized attributes and levels, the researchers discovered a set of combinations that were never explored by past studies. The findings could be maximized by martial arts schools’ stakeholders in streamlining business operations. This study concluded that martial artists had the most preference towards the 6^th^ stimulus with the following combination: MMA training, competition goal, open class, once or twice a week schedule, 0 to 8 km distance between residence and training center, 300,000 to 450,000 IDR (21 to 30 USD) monthly cost, and no gender nor age preferences. Martial artists preferred engaging in MMA competitions if they would be trained with huge and diverse groups in a nearby training center that offered an average price range. MMA competitors would see value for money if they were trained by the finest coaches in the nearest training center with a suitable environment [8]. They felt confident in attending training once or twice a week as long as the conditioning programs included sparring with other gym members. They also believed that training with non-specified demographics would help them enhance their techniques and improve reflexes [1, 6, 11]. On one hand, the martial artists’ least preferred stimulus was the 21^st^ combination: Karate training, competition goal, private class, once or twice a week schedule, >15 km distance between residence and training center, >450,000 IDR (at least 31 USD) monthly cost, and same age preferences. It was the least preferred stimulus given that most attribute levels had negative utility estimates. Karate competitors preferred private classes because their coaches were portrayed as father figures whom they could rely on [11]. It also showed their consciousness of interacting with people because they preferred attending training programs of the same age. Most likely, they wished to attend private classes with their close peers. These martial artists least preferred traveling for at least 41 minutes, as this could be extended depending on Indonesia’s traffic conditions. Moreover, a lot of respondents were relatively young and lacked the financial freedom to purchase the highest package cost.

### 4.2 Managerial implications

This research aims to improve the business operations of martial art schools through the application of conjoint analysis. Based on the findings, it was revealed that training centers should put importance on the type of martial arts, location/distance, and price range. These top three attributes must be considered by martial art schools upon restructuring their internal operations. Considering the results of past studies in similar contexts, none of them found the criticality of these attributes [21–23]. Hence, the presented findings validated the study’s objectives and maximized novel methods.

There were several types of martial arts and schools should offer Muay Thai or Kickboxing, MMA, Taekwondo, Karate, and Boxing throughout the weekly schedule. Training centers should find credible coaches who could spearhead these martial arts. People would only enroll at a martial arts school once their preferred martial art was offered and would immediately look for another school if their preference was unavailable. The schools could divide daily schedules into different sets of martial arts. For example, Muay Thai or Kickboxing could be offered every Friday evening and Sunday morning and MMA could be facilitated every Thursday evening and Saturday morning. There should be no overlapped training periods to accommodate the needs of martial artists. The schedule should be customized depending on the majority of the participant’s availability or the target market’s preference. This approach would attract all types of martial artists and increase school members. It would enhance the probability of getting customers since they could choose a variety of training programs in one training center.

While the distance was a non-negotiable factor for martial arts schools that had already started operating, these schools should highlight types of martial arts instead. But with the help of the government, the road infrastructure must be fixed to minimize traffic and increase mobility. Meanwhile, businessmen who wished to put up a school should perform market research before establishing a martial arts training center. The location must be within 0 to 8 km of the target market’s areas. If the target market was unclear, businessmen should put up the school within residential, university, and office areas. For instance, the west side of Surabaya, Indonesia’s main street could be explored because it was strategically positioned to access nearby cities. This circumstance would manifest easy travel and diverse martial artist leads.

Furthermore, the training schools should ensure that their monthly packages would be priced at most 450,000 IDR (≤ 30 USD). Expensive training programs would limit potential targets to join the martial arts school. Thus, it would be an advantage if the schools could offer 150,000 to 300,000 IDR (10 to 20 USD) as this was the most preferred price range. An affordable price was equated to customer retention and an increase in sales. Martial arts schools should focus on reducing overhead expenses to minimize training cost packages. Particularly, non-value-added activities (labor and materials) should be eliminated and business processes must be streamlined to ensure efficiency.

Although goal attribute was among the least priorities, the top stimulus accentuated the importance of participating in MMA competitions. The regular conditioning programs only included sparring sessions with a coach or other training school members. Thus, the researchers recommended organizing competitions within the training centers. Martial artists’ abilities must be recognized in a formal competition where professional scoring and awards were presented. This technique would increase the engagement of martial arts school members in attending more classes. Martial arts schools may record the competition and provide live streams to multiple social media platforms because the videos might reach potential leads to join the martial arts school.

### 4.3 Contributions to science

The findings provided a scientific expansion of conjoint analysis to sports branding and market research. Specifically, the statistical approach of conjoint analysis designed the rankings of martial arts schools’ attributes and levels. While past studies utilized conjoint analysis in the physical fitness industry, they overlooked a specific sport [21–23]. None of the past studies found that the most important attribute was the type of martial arts, followed by distance, and price range. The present study supported the combination of these most important attributes alongside other essential attributes, such as social environment, class preference, goal, and schedule. The science of ranking attributes and their underlying levels promotes further understanding and new knowledge through the application of advanced statistical techniques. In addition, the study contributed 21 unique stimuli. This scientific approach was never discussed by other researchers [18, 23]. The stimuli were also supported by credible data by incurring a significance value of 0.001. Since this is the first academic research focusing on conjoint analysis and the martial arts industry, a 0.05 standard p-value could be improved by considering a 0.001 significance value instead. Therefore, a new significance value benchmark could be used to ensure higher accuracy.

### 4.4 Limitations and future research

The researchers achieved the study’s objective and presented influential findings about Indonesian martial artists’ preferences in choosing a martial arts school. Nevertheless, the authors acknowledged the limitations that could help other researchers expand the context. First, the researchers gathered a total of fifty-five (55) martial artists, which was deemed a small sample size compared to other experimental studies. This limitation occurred because the researchers looked for respondents residing in Surabaya, Indonesia. Second, the age of the respondents was dominated by 21 to 25 years old. A wider range of ages would result in a better understanding of preferences across different generations. These first two limitations could be mitigated by looking for martial artists through social media platforms. Online platforms could bring opportunities to look for respondents outside the common urban areas. Nevertheless, the current study’s data collection was considered satisfactory because conjoint analysis assured results reliability regardless of the sample size and demographic characteristics [18]. Third, the researchers analyzed twenty-one (21) stimuli, and a more comprehensive analysis could be generated if the stimuli were increased. But future researchers should find the highest number of stimuli that would not make the respondents feel fatigued. Answering lots of items might jeopardize the quality of responses and the interest of participants. Particularly, other researchers may perform multiple testing stages prior to the actual questionnaire distribution. Although this limitation was present, the study applied the formula of Hair et al. [32]. Thus, the researchers should investigate a minimum of 17 stimuli, which was achieved in the present research. Lastly, the study’s market segmentation was underexplored. The researchers focused on martial artists’ preferences more than the sales area of the martial arts school. Although the researchers suggested improvements to enhance martial arts school operations, future researchers could expand the study by incorporating cluster analysis. For instance, k-means clustering could complement conjoint analysis to delve deeply into the martial artists’ preferences once they were grouped accordingly.

## 5. Conclusion

The study analyzed the preferences of martial artists towards martial arts schools’ business approach through conjoint analysis. The importance of 7 attributes was organized from the most important to the least important attribute accordingly: type of martial arts, distance, price range, social environment, class preference, goal, and schedule. The top two martial arts that must be prioritized were Muay Thai or Kickboxing and MMA. In addition, the martial arts schools should be located within 15 km, but at most 8 km would entice more practitioners. Most martial artists would value training programs worth 150,000 to 300,000 IDR (10 to 20 USD). As regards the social environment, martial artists did not mind training with different genders and ages. Moreover, martial artists preferred attending open classes and joining huge groups compared to a one-on-one session with the coach. It was also noted that most respondents treat martial arts as a hobby. Lastly, the optimal training schedule was attending one to two times a week. On the other hand, twenty-one (21) stimuli with different sets of attribute levels were assessed. The most preferred combination was MMA type of martial arts, competition goal, open class, once or twice a week schedule, 0 to 8 km distance, 300,000 to 450,000 IDR (21 to 30 USD) monthly training price, and no social environment preferences. Through the presented findings, the researchers suggested practical applications that would help martial arts schools increase revenues and maintain market competitiveness. Finally, this research contributed to the academe since it was perceived to be one of the pioneer studies focusing on martial artists’ segmentation and martial arts schools’ marketing progression.
